# A comprehensive binding and neutralization analysis of plasma of HIV-1 subtype-C infected donors from India suggest MPER directed neutralization

**DOI:** 10.1186/1742-4690-9-S2-P61

**Published:** 2012-09-13

**Authors:** R Andrabi, M Bala, R Kumar, K Luthra

**Affiliations:** 1 All India Institute of Medical Sciences (AIIMS), New Delhi, India; 2 Regional STD Teaching Training & Research Centre, Safdarjang Hospital, New Delhi, India; 3 AIIMS, New Delhi, India

## Background

Dissecting the specificities of the anti-HIV-1 neutralizing antibodies will assist in identifying targets for an HIV-1 subunit vaccine which undoubtedly remains ultimate goal for vaccine development.

## Methods

We tested 30 HIV-1 drug naive plasma samples for neutralization against a broad panel of subtype-A, B and C tier 1 and tier 2 viruses in a TZM-bl assay. Three broadly neutralizing plasma (bNP) samples (AIIMS206, AIIMS239 and AIIMS249) were tested for ELISA binding with a set of 211 consensus-C gp160 overlapping peptides (NIH AIDS Research and Reference Reagent Program). The competition and depletion experiments (for neutralization) were carried out with peptides corresponding to consensus-C V3 (35mer), IDR (19mer) and MPER (24mer).

## Results

Approximately 25% of the plasma/virus combinations showed neutralizing activity with a predominance of subtype C specific neutralization compared to subtype B (p=0.001). Immunoglobulin-G fractions from bNP were shown to mediate neutralization exclusively and were shown to retain the binding to subtype-A, B and C recombinant gp120 proteins. Based on the Max50 ELISA binding titers, the immunoreactivity of the three bNP mapped to second variable (V2), second constant (C2), third variable (V3), fourth constant-fifth variable (C4-V5), fifth constant (C5) regions of gp120 and fusion protein (FP), immunodominant region (IDR), C-heptad region (CHR), membrane proximal external region (MPER) and C-terminal (CT) of gp41 protein. In the depletion and competition assays, the bNP AIIMS206 and AIIMS239 showed dependence on MPER directed antibodies with four and six (out of eight) viruses respectively while the V3 and IDR peptides showed minimal effect on neutralization as compared to untreated and mock depleted plasma controls. Further, the mapping of IgG fractions from bNP with overlapping MPER peptides showed 2F5 like binding specificity for AIIMS206 plasma.

## Conclusion

Our study demonstrates that MPER directed antibodies in HIV-1 subtype-C infected patients play a crucial role in viral neutralization.

